# Effects of Different Doses of Eucalyptus Oil From *Eucalyptus globulus* Labill on Respiratory Tract Immunity and Immune Function in Healthy Rats

**DOI:** 10.3389/fphar.2020.01287

**Published:** 2020-08-21

**Authors:** Jie Shao, Zhenjie Yin, Yaqin Wang, Yudong Yang, Qing Tang, Mingming Zhang, Jieying Jiao, Chengjie Liu, Mingfang Yang, Lifang Zhen, Amira Hassouna, William Lindsey White, Jun Lu

**Affiliations:** ^1^521 Hospital of Norinco Group, Xi’an, China; ^2^School of Public Health and Interdisciplinary Studies, Faculty of Health and Environmental Sciences, Auckland University of Technology, Auckland, New Zealand; ^3^Department of Medical Biochemistry and Molecular Biology, Faculty of Medicine, Cairo University, Cairo, Egypt; ^4^School of Science, Faculty of Health and Environmental Sciences, Auckland University of Technology, Auckland, New Zealand; ^5^College of Food Engineering and Nutritional Science, Shaanxi Normal University, Xi’an, China; ^6^Maurice Wilkins Centre for Molecular Biodiscovery, Auckland, New Zealand

**Keywords:** eucalyptol, respiratory tract, immunity, NK cells, macrophages, CD4/CD8, T/B cells, immunoglobulins

## Abstract

Eucalyptol (1,8-cineole), the major constituent of eucalyptus oil (EO), was used in traditional medicine as a remedy for colds and bronchitis. This study aimed at clarifying the effect of eucalyptol on respiratory immune function of CD8 and CD4 cells, and alveolar macrophages (AM). Thirty male Sprague-Dawley rats were divided into experimental and control groups. The drug was given once a day for 3 weeks and the experimental group was divided according to the eucalyptol dose into: 30, 100, and 300 mg·kg^-1^ groups. Flow cytometry was used to detect the phagocytic function of CD4, CD8 cells, and AM in the bronchopulmonary lavage fluid. The 30 and 100 mg·kg^-1^ groups had an up-regulation effect on CD8 (p < 0.05), with no significant effect on macrophage phagocytosis. The 300 mg·kg^-1^ group had an inhibitory effect on CD8 and macrophage phagocytosis (p < 0.05), with no significant difference in CD4 between groups. Further investigation was conducted to evaluate the effect of EO on immune function in rats by detecting blood T, B, and NK cells using flow cytometry, and blood IgA, IgG, IgM, and IFN-γ levels by ELISA. High dosage of eucalyptol significantly reduced the proportion of blood B and NK cells (p < 0.05). IgA was decreased in the 100 and 300 mg·kg^-1^ groups (p < 0.05). There are no significant differences between the number of T cells and the IgG, IgM, and IFN-γ levels between experimental and control groups. Rational use of EO containing eucalyptol can improve the immune function of the respiratory tract and the body immunity, while high dose could have damaging effects, through modifying the phagocytic function of CD8 cells and reducing the proportion of blood B cells, NK cells, and IgA.

## Introduction

Inflammatory conditions are known to reduce both productivity and quality of life, causing immense financial losses (de Cássia da Silveira e Sá et al., 2013). Synthetic steroidal and non-steroidal anti-inflammatory drugs and antibiotics are commonly used for treating the increasingly prevalent inflammatory and infectious conditions. However, they possess undesirable health consequences and side effects (Sarkar et al., 2016). Alternative safe therapeutic interventions from natural sources are in demand in general populations (Yadav and Chandra, 2017). Clinical and pre-clinical studies exploring essential oils and their chemical ingredients are increasing (de Cássia da Silveira e Sá et al., 2013). *Eucalyptus globulus*, commonly known as blue gum tree, is a species of tall, evergreen tree. It has a wide distribution in China, and trees in Yunnan Province in particular has a high content of 1,8-cineole (more than 70%) in leaves, commercially used for the production of essential oils in the pharmaceutical and cosmetic industries (Dhakad et al., 2018), which has rich ethanomedicinal and therapeutic importance (Kesharwani et al., 2018). It is an aromatic medicinal plant with main pharmacological constituent 1,8-cineole (Yadav and Chandra, 2017) also known as eucalyptol (de Cássia da Silveira e Sá et al., 2013). Independent studies have reported anti-inflammatory properties of eucalyptus oil (EO) and its principle component eucalyptol (1,8- cineole) (Santos and Rao, 2000; Silva et al., 2003; Sadlon and Lamson, 2010; Yadav and Chandra, 2017), which, depending on the source species of eucalyptus, accounts for up to 75% of eucalyptus oil’s content (Aziz et al., 2019). Essential oils of various eucalyptus species are utilized in the cosmetic, food, and pharmaceutical industries (Marzoug et al., 2011). Studies show that EO containing eucalyptol has a strong broad-spectrum antibacterial effect against many microorganisms in clinical settings (Hendry et al., 2009). EO also possesses potent antiseptic, astringent, diaphoretic, deodorant, inhalant, expectorant, insect repellent, and suppurative properties (Fresquet Febrer, 1995; Javaid and Samad, 2012).

Many respiratory diseases directly result from or are associated with erratic immunological responses (Chen and Kolls, 2013), while EO appears to be able to modulate those responses. Natural killer (NK) cells are a crucial component of the innate immune system, which can destroy tumor cells without previous sensitization, as well as the production of immunoregulatory cytokines early in an immune response (Trinchieri, 1989; Biron et al., 1999), particularly interferon-γ (IFN-γ) and tumor necrosis factor-α (TNF) (Yokoyama, 1999). Besides their direct effect in early immune responses, all of these cytokines are important modulators of the subsequent adaptive immune response mediated by T and B cells (Nakamura, 2002). Cytotoxic T cells phagocytose pathogens. B cells produce and secrete antibodies or immunoglobulins (Ig), stimulating the immune system to destroy the pathogens. This collaboration underlies the development of optimal antibody responses, permitting the blockade of viral cell entry and the rapid neutralization of bacterial toxins which is required for host defense (Marshall and Swain, 2011; Petersone et al., 2018).

The beneficial use of EO, based on its anti-inflammatory and anti-oxidative properties, has been demonstrated in clinical trials to act as long-term therapy in the improvement of asthma control and prevention of chronic obstructive pulmonary disease (COPD)-exacerbations (Juergens, 2014), as well as improving mucous membrane ciliary movement and having broncho-spasmolytic and secretolytic properties (Wittmann et al., 1998). However, there is a shortage of knowledge on the mode of action of EO (Yadav and Chandra, 2017). The induction of p38 MAPK dephosphorylation may be one of the common mechanisms by which citrine regulates mucin secretion and anti-inflammatory effects. It is believed that EO plays a major role in this pathway (Sarkar et al., 2016). Juergens and colleagues found that EO can inhibit the release of PGE2 and LTB4 from peripheral blood mononuclear cells in asthmatic patients, and inhibits TNF-α, IL-1, LTB4, and thromboxane B2 in peripheral blood monocytes stimulated by lipopolysaccharide (LPS) in healthy people (Juergens et al., 2003). Eucalyptol acts as an anti-inflammatory modifier by controlling inflammatory processes and mediates production of infection rather than acting as a simple mucolytic agent. Macrophages and monocytes are the most affected white blood cells, with increased phagocytic activity (Sadlon and Lamson, 2010). However, in another study, EO pre-treatment did not significantly enhance the phagocytic activity but significantly enhanced the pathogen clearance. Pre-treatment of EO has diminished inflammatory response and attenuated LPS-induced inflammatory signaling pathways at various levels (Yadav and Chandra, 2017).

It thus appears that more details behind these effects need to be clarified. This study aims to explore the effect of EO on the respiratory function of rats to provide a theoretical basis to clarify the effect of the drug on immune function of CD8, CD4 cells, and alveolar macrophages (AM), through analysis of blood T cells, B cells, and NK cells by flow cytometry, and blood IgA, IgG, IgM, and IFN-γ levels by ELISA.

## Materials and Methods

### Experimental Animal and Administration Method

Thirty Sprague-Dawley male rats weighing 200–250 g were purchased from Experimental Animal Center of Xi’an Jiaotong University (Xi’an, Shaanxi Province, China). The experimental design was similar to a previous study, including dosage and period of treatment (Lü Xiaoqin et al., 2004). Institutional animal ethics approval was gained prior to animal experiment (approval number 2016SF-154f). Rats were randomly distributed into four experimental groups, including eight rats each in low, medium, and high dose groups (30, 100, and 300 mg·kg^-1^ of eucalyptol, dose was in eucalyptol, i.e. 30 mg·kg^-1^ was 36.1 mg·kg^-1^ EO), and six in the control group (canola oil used for dissolving eucalyptol was administered). EO or canola oil was administered intra-gastrically once a day for 3 weeks, at the end of which, the left and right main bronchi were separated, and bronchopulmonary lavage was performed separately. Below procedures were carried out to ensure no contamination from circulating blood. Trachea was exposed surgically, and the right primary bronchus was clamped. A tube was inserted to the left primary bronchus, and 2 ml saline (0.025 g·ml^-1^) was used to flush alveoli and retrieved. The retrieved volume is around 1.5 ml. The flush was repeated three times. Then, same procedure was applied to the right primary bronchus. The retrieved fluids are mixed and combined for the final experiment. Before bronchi collection, 4 ml of heparinized blood was taken *via* cardiac puncture for testing.

### Main Reagents and Equipment

Extracted EO containing 83% of eucalyptol in canola oil was a gift from Beijing Jiuhe Pharmaceutical Co. Ltd. (Beijing, China). It was a light-yellow volatile liquid, and insoluble in water. Red blood cell lysate, *E. coli* preparation (5 × 10^8^/ml), Percoll separation solution, CD3, CD4, CD8, CD19, and CD56 monoclonal antibodies, allophycocyanin (APC), phycoerythrin (PE), Fluorescein (FITC), goat anti-Mouse IgG (H+L), and sIgA detection kit were purchased from Thermo Fisher Scientific (Waltham, MA, USA). FACS hemolysin and paraformaldehyde were purchased from Wuhan Booute Biotechnology Co. Ltd. (Wuhan, Hubei Province, China).

A Cytomics FC 500 flow cytometer from Beckman Coulter Inc. (Brea, CA, USA), an ELX800TM plate-reader from Biotek Instruments Inc. (Winooski, VT, USA), and a Forma 381 CO_2_ incubator from Thermo Fisher Scientific were used in the experiments.

### Methods

#### Detection of CD4 and CD8 in Bronchoalveolar Lavage Fluid

After isolating the AM in the lavage fluid, its concentration was adjusted to approximately 1 × 10^6^/ml, and inoculated into a petri dish with a 10 cm diameter. It was incubated at 37°C for 2 h in a CO_2_ incubator. Non-adherent cells were washed away with D-Hank’s. A bacterial solution of 200 μl was added to each sample (2 ml) and incubated at 37°C for 1 h. Then, 200 μl of the phagocytic bacterial liquid was added, and CD4 and CD8 monoclonal antibodies were added, respectively, and incubated at 4°C for 1 h in the dark. Two ml of FACS hemolysin was added, and the mixture was allowed to stand in the dark for 10 min. It was then centrifuged at 1,500 rpm for 5 min, the supernatant discarded, and 2 ml PBS was added. The mixture was then centrifuged at 1,500 rpm for 5 min, the supernatant discarded, and 2 μl paraformaldehyde solution added for flow cytometry analysis.

#### Determination of Phagocytosis of Alveolar Macrophages

The lavage solution was transferred to a 50 ml centrifuge tube, centrifuged at 1,500 rpm for 5 min, and the pellet was resuspended in RPMI 1640 containing 10% fetal bovine serum with AM at a concentration of 1 × 10^6^/ml and inoculated onto a Petri dish. It was incubated at 37°C for 2 h, after which non-adherent cells were washed away with D-Hank’s. A bacterial solution of 200 μl was added to each sample and incubated at 37°C for 2 h. Flow cytometry was used to detect phagocytosis.

#### Detection of Blood T Cells, B Cells, and NK Cells

CD3, CD3/CD19 goat anti-Mouse IgG, CD3/CD56 goat anti-mouse IgG were added to a 200 μl sample and incubated at 4°C for 1 h in the dark. Two ml of FACS hemolysin was added; the mixture was allowed to stand in the dark for 10 min, and then centrifuged at 3,000 rpm for 5 min. The supernatant was discarded, 2.0 ml of PBS were added, centrifuged at 3,000 rpm for 5 min, with the supernatant discarded, and 100 μl of 2% paraformaldehyde solution were added for flow cytometry analysis. T cells, B cells, NK cells, and other white blood cells were detected and counted by the flow cytometer and percentages T cells, B cells, and NK cells in total white blood cells were calculated by the machine automatically.

#### Detection of Blood IgA, IgG, IgM, IFN-γ

IgA, IgG, IgM, and IFN-γ in samples were measured according to the ELISA kit manufacturer’s instruction using a microplate reader for final colorimetric reading.

### Statistical Analysis

Data was checked for a normal distribution first and then a one-way analysis of variance (ANOVA) was performed with least significant difference (LSD) *post-hoc* test to analyze differences between groups using SPSS Statistics software (IBM, Armonk, NY, USA). A P value of <0.05 was considered statistically significant.

## Results

### CD4, CD8, and AM in Alveolar Lavage Fluid

**Figure 1:**
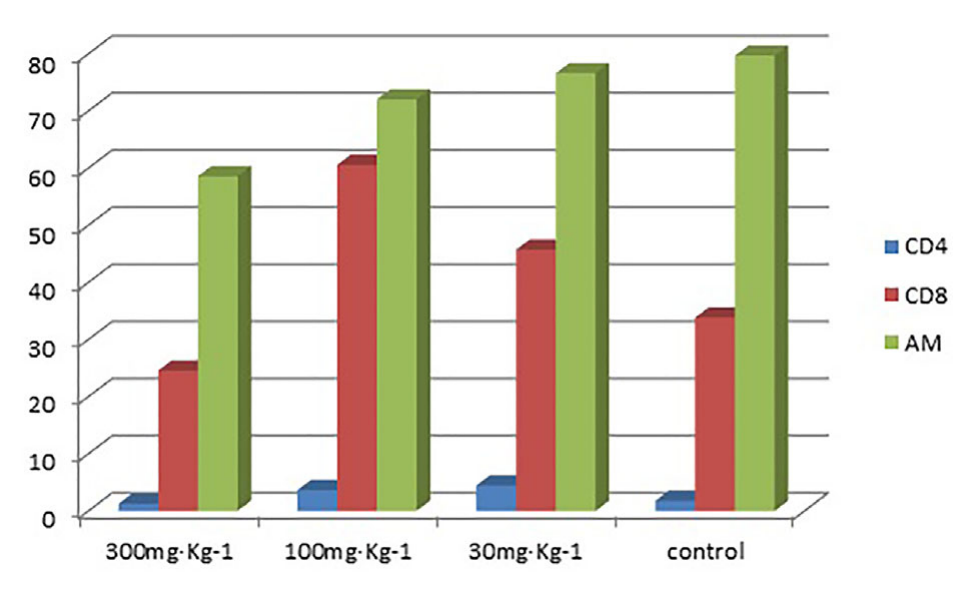
Percentages of CD4 and CD8 in lymphocytes and levels of *AM* phagocytosis in alveolar lavage fluid.

The results in **Table 1** show that the high-dose group had an inhibitory effect on CD8 in alveolar lavage fluid (P < 0.05). The medium-dose group and the low-dose group had an up-regulation effect on CD8 in alveolar lavage fluid (P < 0.05) (**Tables 1** and **2**). The high-dose group had an effect on macrophage phagocytosis (P < 0.05, **Figure 1**). There was no significant difference between the middle dose, low dose, and control group (P > 0.05) (**Table 3**). There was no significant difference between the CD4 in the lavage fluid and the control group (P > 0.05), indicating that eucalyptol had no significant effect on CD4 in the blood alveolar lavage fluid of rats.

**Table 1:** Percentages of CD4 and CD8 in lymphocytes and levels of AM phagocytosis in alveolar lavage fluid.

Dose of eucalyptol	300 mg·kg^-1^ group	100 mg·kg^-1^ group	30 mg·kg^-1^ group	Control group	Comparison between groups
CD4	1.2 ± 0.9	3.4 ± 2.1	3.5 ± 1.7	1.8 ± 0.8	P > 0.05
CD8	24.6 ± 12.8	60.6 ± 4.5	45.7 ± 14.7	33.8 ± 1.7	P < 0.05
AM	58.6 ± 14.8	72 ± 6.2	76.6 ± 7.9	79.7 ± 0.3	P < 0.05

**Table 2:** Comparison of CD8 in different groups.

	Group(I)	Group(J)	Mean difference(I–J)	Standard error	P value	95% Confidence interval	
						Lower limit	Upper limit
LSD	1	2	−36.0*	8.76	.005	−56.7	−15.3
		3	−21.2*	8.76	.046	−41.9	−.46
		4	−9.27	9.79	.375	−32.4	13.9
	2	1	36.0*	8.76	.005	15.3	56.7
		3	14.8	8.76	.134	−5.87	35.5
		4	26.7*	9.79	.029	3.58	49.9
	3	1	21.2*	8.76	.046	.46	41.9
		2	−14.8	8.76	.134	−35.5	5.87
		4	11.9	9.79	.264	−11.3	35.1
	4	1	9.27	9.79	.375	−13.9	32.4
		2	−26.7*	9.79	.029	−49.9	−3.58
		3	−11.9	9.79	.264	−35.1	11.3

1–4 correspond to 300 mg·kg^-1^, 100 mg·kg^-1^, 30 mg·kg^-1^, and control group, respectively. *P < 0.05. Group I and J were automatically assigned by SPSS. Group I is the group to be compared with, while Group J contains the ones that are used to compare against Group I.

**Table 3:** Comparison of AM in different groups.

	Group(I)	Group(J)	Mean difference(I–J)	Standard error	P value	95% Confidence interval	
						Lower limit	Lower limit
LSD	1	2	−13.4	7.79	.128	−31.9	4.97
		3	−18.1	7.79	.053	−36.5	.35
		4	−21.1*	8.71	.046	−41.7	−.52
	2	1	13.4	7.79	.128	−4.97	31.9
		3	−4.62	7.79	.572	−23.1	13.8
		4	−7.67	8.71	.408	−28.3	12.9
	3	1	18.1	7.79	.053	−.35	36.5
		2	4.62	7.79	.572	−13.8	23.0
		4	−3.05	8.71	.737	−23.6	17.5
	4	1	21.1*	8.71	.046	.52	41.7
		2	7.67	8.71	.408	−12.9	28.3
		3	3.05	8.71	.737	−17.5	23.6

See legend in **Table 2**. *P < 0.05.

### Flow Cytometry for Detection of Blood T Cells, B Cells, and NK Cells

**Figure 2:**
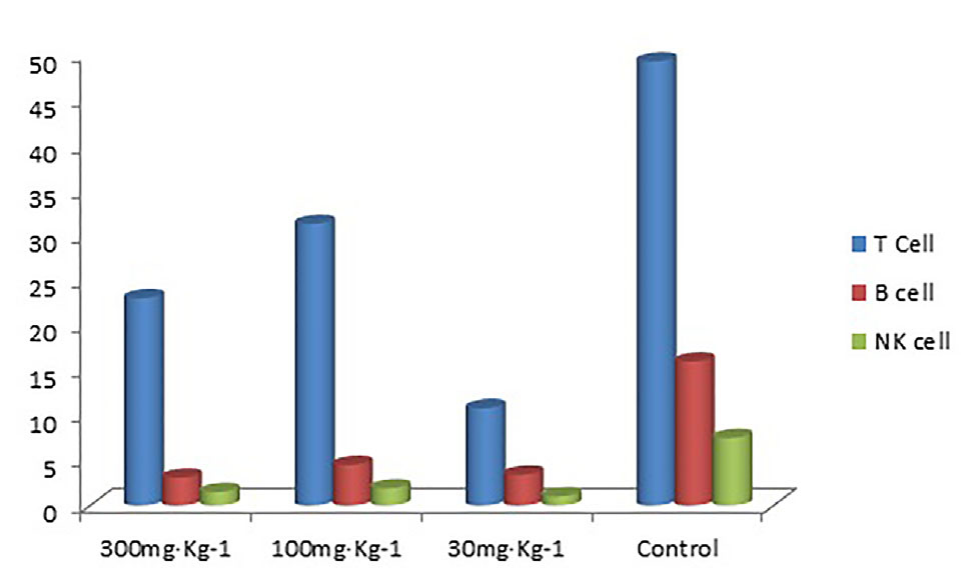
T cell, B cell, and NK cell (%) in rat blood.

The results in **Tables 4**–**6**) show that EO significantly decreased the proportion of B cells and NK cells in blood, and there was a significant difference between the drug groups and the control group (P < 0.05). There was no significant difference between the drug groups with different doses (P > 0.05), indicating that eucalyptol had an inhibitory effect on blood B and NK cells in rats regardless of dose (**Figure 2**). There was no significant difference in the proportion of T cells in the blood between the drug groups and control group (P > 0.05), indicating that EO had no significant effect on the proportion of blood T cells in rats.

**Table 4:** T cell, B cell, and NK cell (%) in blood.

*GroupType of cells*	300 mg·kg^-1^ group	100 mg·kg^-1^ group	30 mg·kg^-1^ group	Control group	Comparison between groups
*T cell*	22.9 ± 13.1	31.2 ± 22	10.8 ± 9.2	49.1 ± 3.3	P > 0.05
*B cell*	3.1 ± 2.1	4.5 ± 2.4	3.4 ± 0.5	15.9 ± 4.3	P < 0.05
*NK cell*	1.5 ± 0.8	1.9 ± 1.4	1.0 ± 0.6	7.5 ± 0.2	P < 0.05

**Table 5:** Comparison of B cells between groups.

	Group(I)	Group(J)	Mean difference (I–J)	Standard error	P value	95% Confidence interval	
						Lower limit	Upper limit
LSD	1	2	−1.41	1.92	.484	−5.95	3.12
		3	−.343	1.92	.863	−4.88	4.19
		4	−12.8*	2.14	.001	−17.9	−7.77
	2	1	1.41	1.92	.484	−3.11	5.95
		3	1.07	1.92	.593	−3.46	5.61
		4	−11.4*	2.14	.001	−16.5	−6.36
	3	1	.343	1.92	.863	−4.19	4.88
		2	−1.07	1.92	.593	−5.61	3.46
		4	−12.5*	2.14	.001	−17.6	−7.43
	4	1	12.8*	2.14	.001	7.77	17.9
		2	11.4*	2.14	.001	6.36	16.5
		3	12.5*	2.14	.001	7.43	17.6

See legend in **Table 2**. *P < 0.05.

**Table 6:** Comparison of NK cells between groups.

	Group(I)	Group(J)	Mean difference (I–J)	Standard error	P value	95% Confidence interval	
						Lower limit	Upper limit
LSD	1	2	−.417	.656	.546	−1.97	1.14
		3	.477	.656	.491	−1.08	2.03
		4	−5.98*	.734	.000	−7.72	−4.25
	2	1	.417	.656	.546	−1.14	1.97
		3	.893	.656	.216	−.659	2.45
		4	−5.57*	.734	.000	−7.30	−3.83
	3	1	−.477	.656	.491	−2.03	1.08
		2	−.893	.656	.216	−2.45	.659
		4	−6.46*	.734	.000	−8.19	−4.72
	4	1	5.98*	.734	.000	4.25	7.72
		2	5.57*	.734	.000	3.83	7.30
		3	6.46*	.734	.000	4.72	8.19

See legend in **Table 2**. *P < 0.05.

### Detection of Blood IgA, IgG, IgM, and IFN-γ Levels in All Groups

**Figure 3:**
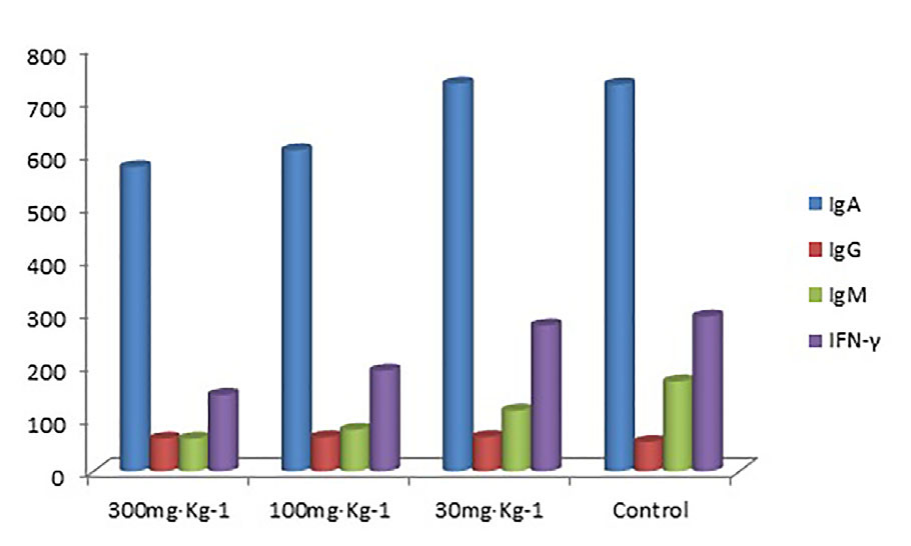
Blood IgA, IgG, IgM, and IFN-γ levels (ng/ml) in all groups.

The blood IgA levels of the high and medium dose groups were significantly lower than the control group (P < 0.05, **Tables 7** and **8**), and there was no significant difference between the low dose and control group (P > 0.05). There was no significant difference in IgG, IgM, and IFN-γ levels between the drug groups and control group (P > 0.05), indicating that EO had no significant effect on IgG, IgM, and IFN-γ (**Figure 3**)

**Table 7:** Blood IgA, IgG, IgM, and IFN-γ levels in all groups.

Type of Ig	300 mg·kg^-1^ group	100 mg·kg^-1^ group	30 mg·Kg^-1^ group	Control group	Comparison between groups
IgA	573.4 ± 31.1	605.1 ± 76.5	730.9 ± 28.4	729.4 ± 41.3	P < 0.05
IgG	61 ± 1.8	63.7 ± 3.9	64.1 ± 5.6	54.7 ± 0.4	P > 0.05
IgM	60.9 ± 7.3	77.9 ± 3.7	114.2 ± 51.4	169.2 ± 104.7	P > 0.05
IFN-γ	143.8 ± 6.8	189.2 ± 15.7	274.5 ± 29.9	291.5 ± 8.8	P > 0.05

**Table 8:** Comparison of IgA levels between groups.

	Group(I)	Group(J)	Mean difference (I–J)	Standard error	P value	95% Confidence interval	
						Lower limit	Upper limit
LSD	1	2	−32.0	40.4	.454	−127.4	63.4
		3	−157.7*	40.4	.006	−253.1	−62.2
		4	−156.2*	45.1	.011	−262.9	−49.5
	2	1	32.0	40.4	.454	−63.4	127.4
		3	−125.7*	40.4	.017	−221.1	−30.2
		4	−124.2*	45.1	.028	−230.9	−17.5
	3	1	157.7*	40.4	.006	62.2	253.1
		2	125.7*	40.4	.017	30.2	221.1
		4	1.5	45.1	.974	−105.2	108.2
	4	1	156.2*	45.1	.011	49.5	262.9
		2	124.2*	45.1	.028	17.5	230.9
		3	−1.5	45.1	.974	−108.2	105.2

See legend in **Table 2**. *P < 0.05.

## Discussion

It is suggested that EO, with its dominant ingredient eucalyptol, might be beneficial to cell mediated immunoregulatory agents in infectious diseases, immunosuppressive pathologies, or after cancer therapy (Sadlon and Lamson, 2010). In the present study, CD4 and CD8 T cells as well as AM were measured in alveolar lavage fluid in rats given different doses of EO *via* the oral route, once a day for 3 weeks. The results showed that 100 and 30 mg·kg^-1^ dose had an up-regulation effect on CD8 (P < 0.05), which may help the body fight inflammation. However, the high dose (300 mg·kg^-1^) had an inhibitory effect on CD8, which may reduce the body immunity. The high-dose also significantly inhibited the phagocytosis of rat alveolar lavage fluid macrophages (P < 0.05), while the medium (100 mg·kg^-1^) and low (30 mg·kg^-1^) doses had no significant effect on macrophage phagocytosis. There is no significant effect on rat alveolar lavage fluid CD4 cells with EO administration.

The results of this study showed that the high dose of EO had an inhibitory effect on the proportion of B cells and NK cells in the blood (P < 0.05), and no significant effect was detected on the proportion of T cells (P > 0.05). Blood IgA decreased significantly (P < 0.05) in 300 and 100 mg·kg^-1^ groups, and there was no significant change in IgG, IgM, and IFN-γ levels (P > 0.05). This study’s results suggest that the high dose of EO can inhibit the body’s immune function, however, the literature to date showed varied results. For example, Lu and co-workers identified 100 mg·kg^-1^ of EO as the most effective dose that produced significant decrease in severity of bronchitis symptoms, less inflammatory cells infiltration, and reduced airway mucin secretion in rat chronic bronchitis caused by lipopolysaccharide (LPS) **(**Lü Xiaoqin et al., 2004). Similarly, Santos and Rao demonstrated that orally administered EO produced anti-inflammatory and antinociceptive effects and is considerably more potent at a dose of 400 mg·kg^-1^ (Santos and Rao, 2000). The difference between those studies and the current one may be because of the animal model difference. The current study used normal healthy rats while those studies used diseased model (induced asthma etc.).

In another *in vitro* study (Serafino et al., 2008), EO inhibited the stimulated cytokine generation from human monocytes and lymphocytes. In that study, a significant increase in monocytes occurred after 15 days of oral administration of EO, without any effect on monocyte or lymphocytes or granulocyte. A significant increase was detected in CD 25 and CD 44 monocyte surface markers but not granulocytes or lymphocytes (Serafino et al., 2008). The EO dose was found to dependently stimulate phagocytosis by human monocyte derived macrophages (Serafino et al., 2008). These findings are consistent with our *in vivo* findings in healthy rats, where low dose of EO stimulated immune response.

Based on alveolar macrophages, this study has demonstrated the anti-inflammatory and anti-infective properties of EO in a pulmonary inflammation and infection model. This is consistent with a previous study, where phagocytic activity was tested *in vitro* in EO treated and untreated human monocyte derived macrophages (MDMs), as well as *in vivo* in monocytes/granulocytes from peripheral blood of immuno-competent or immuno-suppressed rats (Serafino et al., 2008). EO was found to stimulate MDMs phagmmocytic activity and that eucalyptol may be acting by complement receptor-mediated phagocytosis (Serafino et al., 2008). Implementation of innate cell-mediated immune response mainly involving the peripheral blood monocytes/granulocytes was also observed *in vivo* after EO administration. The combined 5-fluorouracil (5-FU)/EO treatment raised the phagocytic activity of the granulocytic/monocytic system, which was significantly decreased by the chemotherapy and inhibited the 5-FU induced myelotoxicity (Serafino et al., 2008).

Many studies demonstrate that eucalyptol may interfere with production of inflammatory mediators as the underlying mechanism of its mucolytic activity (Juergens et al., 2004). Santos and Rao reported that relatively high concentrations of EO, systemically administered, caused an inhibitory effect on experimental inflammatory conditions in rats with ethanol-induced gastric injury (Santos and Rao, 2001). Our study on healthy rats showed that at high dose, EO inhibited CD8 and macrophage phagocytosis, which is consistent with the above report. Pattnaik and co-workers used EO as an additional therapy in patients with mild and moderate asthma for over 3 days. They observed improvement in lung function, as well as suppression of *ex vivo*-stimulated inflammatory mediator production in short-term cultures of peripheral monocytes (Pattnaik et al., 1997). It is hard to compare our data with this report because EO was used as an additional therapy and any anti-inflammatory effect may not due to EO’s effect alone. Another study indicated that long-term therapy with EO was well tolerated and capable of mediating anti-inflammatory activity equivalent to approximately 3 mg prednisolone, providing new evidence to support long-term systemic therapy with EO for treatment of asthma and COPD (Juergens, 2014). In addition, EO has an inhibitory effect on eosinophils and neutrophil aggregation in ovalbumin-induced asthmatic guinea pig (Zhang and Bevan, 2011). Both above studies indicate that EO has anti-inflammatory effects, consistent with our rat study, where low dose EO administration improve inflammatory related cytokines.

The upregulation effect of EO on CD8 cells found in our study is of particular importance, as CD8 T cells play fundamental roles in immune memory during primary respiratory virus infections (Zhang and Bevan, 2011; Retamal-Díaz et al., 2019). The stimulation of CD8 at low EO dose found in our study provided important indication that eucalyptol may be beneficial in immune cell memory enhancement.

To our knowledge, the present study is the first to explore the effect of different EO doses on Ig. The only Ig that was affected by high doses was IgA. The blood IgA significantly decreased (P < 0.05) in the 300 mg·kg^-1^ groups. IgA levels are higher than other immunoglobulins at mucosal surfaces and in secretions (Woof and Mestecky, 2005). IgA contributes up to 50% of the protein in the “first milk” given by the mother to the neonate. Bacterial or viral infection and pathogenesis are prevented by intracellular IgA, as it is critical at protecting mucosal surfaces by direct neutralization of toxins, bacteria, and virus or by prevention of their binding to the mucosal surface (Stubbe et al., 2000). Medium and high doses of EO clearly reduce IgA in our healthy rat study. The decrease of IgA level may cause undesirable immune-suppression. Therefore, an optimal dose of EO is important to reach the desired beneficial effect.

In conclusion, regulation of immune homeostasis is a delicately and tightly regulated process. The present study reports that high dose of EO can damage the immune function of the respiratory tract and the body immunity, possibly through B cell modulation, which results in an increase of plasma cells, inflammatory intermediates, and immune complexes (Jelinek, 2000; Tellier et al., 2016); while low dose can produce an improving effect, possibly through anti-inflammation, increase of excretion of mucosa, and improvement of airway mucociliary movement (Lü Xiaoqin et al., 2004). Therefore, ensuring appropriate dosage and rational use of EO will be beneficial for improving the immune function, avoiding possible damage to the immune function in general or to the respiratory tract in particular. The specific mechanism of action of such dose differences needs to be further studied. Our experiments provide an *in vivo* basis in a healthy animal model, which will benefit further clarification of the effect of EO (hence eucalyptol) on the immune function.

## Data Availability Statement

The raw data supporting the conclusions of this article will be made available by the authors, without undue reservation.

## Ethics Statement

The animal study was reviewed and approved by the ethics committee of 521 Hospital Ethics Committee.

## Author Contributions

Conceptualization, JS, LZ, JL, and YY. Methodology, JS, ZY, YW, QT, and MZ. Validation, JJ, CL, MY, AH, and JL. Formal analysis, JS, ZY, and JL. Investigation, JS, LZ, ZY, and YW. Resources, JS and LZ. Data curation, JS, ZY, YW, and QT. Writing—original draft preparation, JS, AH, and JL; writing—review and editing, JS, WW, and JL.

## Funding

This research was funded by Shaanxi Province Science and Technology Programme project number 2016sf-154.

## Conflict of Interest

The authors declare that the research was conducted in the absence of any commercial or financial relationships that could be construed as a potential conflict of interest.

## Supplementary Material

Supplement Figure 1 T cell flow cytometer results.

Supplement Figure 2 B cell flow cytometer results.

Supplement Figure 3 CD4 flow cytometer results.

Supplement Figure 4 CD8 flow cytometer results.

Supplement Figure 5 Macrophage phagocytosis flow cytometer results.
