# Supplementary figures and images for: Effects of Different Doses of Eucalyptus Oil From Eucalyptus globulus Labill on Respiratory Tract Immunity and Immune Function in Healthy Rats
Source: Front Pharmacol. 2020 Aug 21;11:1287. doi: 10.3389/fphar.2020.01287 (PMC7472567; doi:10.3389/fphar.2020.01287)

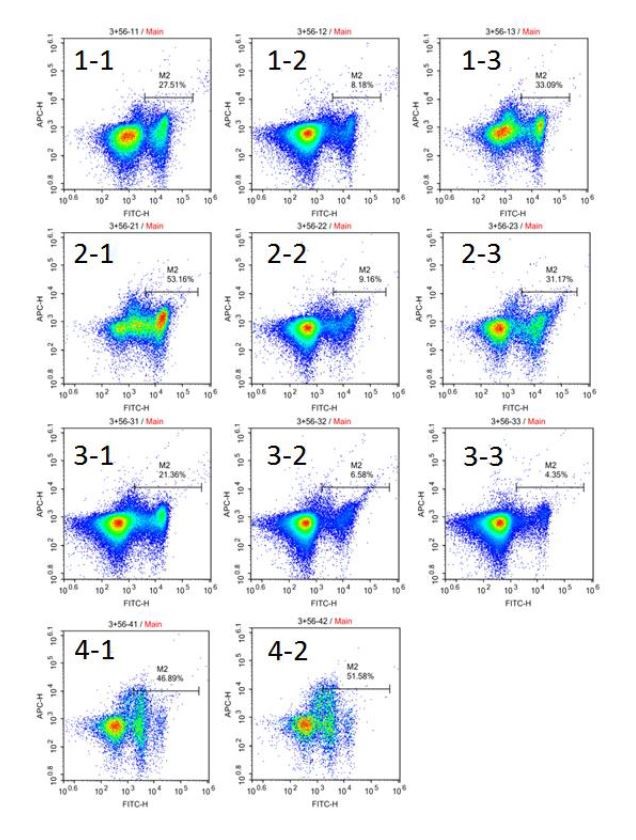

Supplement: Supplement Figure 1 — T cell flow cytometer results. [file Image_1.jpeg]

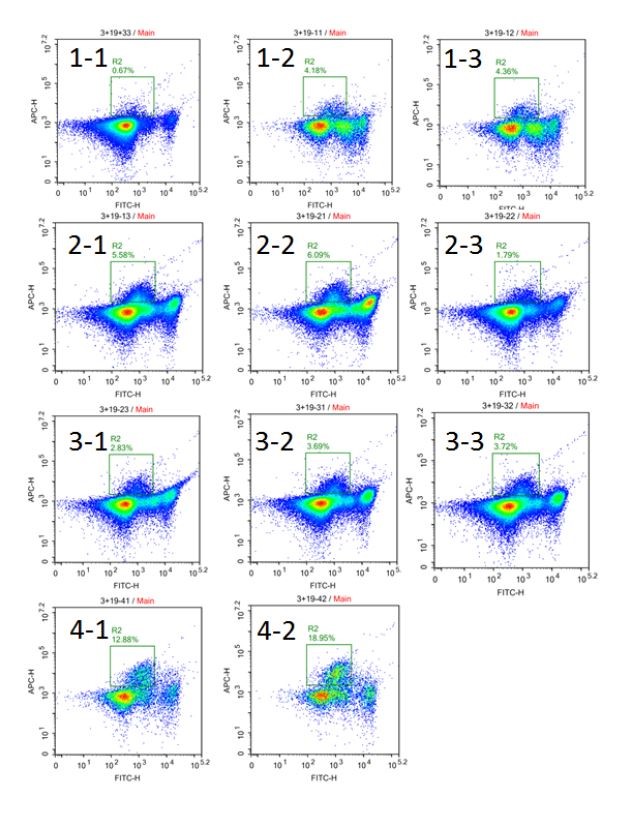

Supplement: Supplement Figure 2 — B cell flow cytometer results. [file Image_2.jpeg]

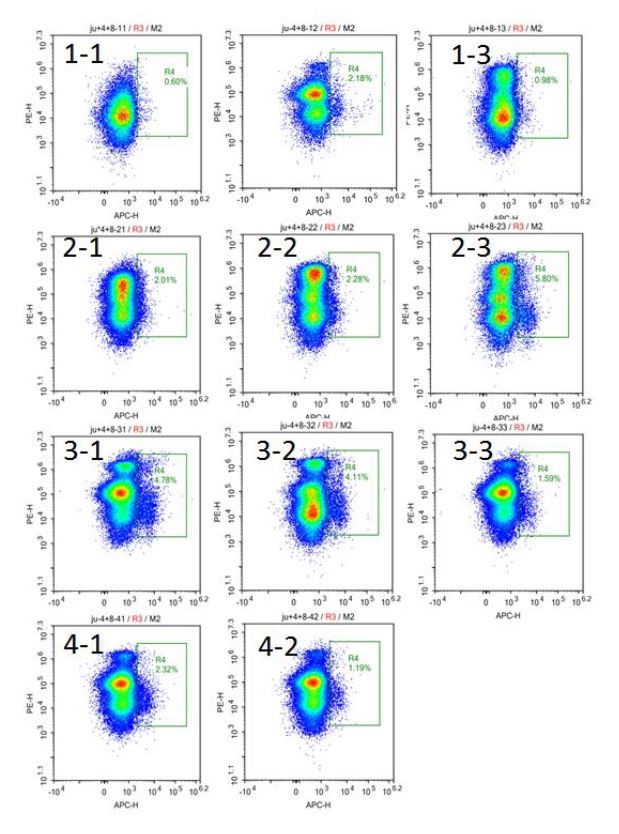

Supplement: Supplement Figure 3 — CD4 flow cytometer results. [file Image_3.jpeg]

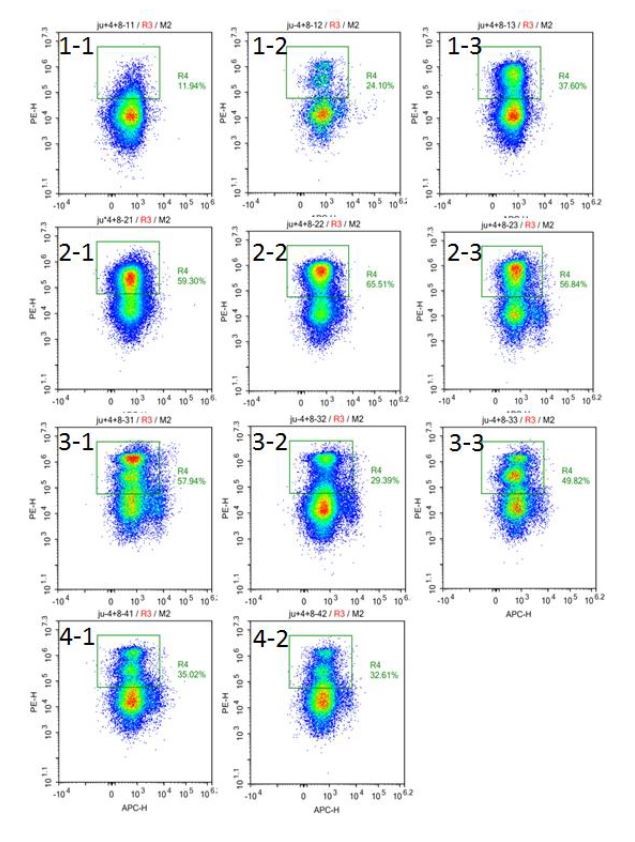

Supplement: Supplement Figure 4 — CD8 flow cytometer results. [file Image_4.jpeg]

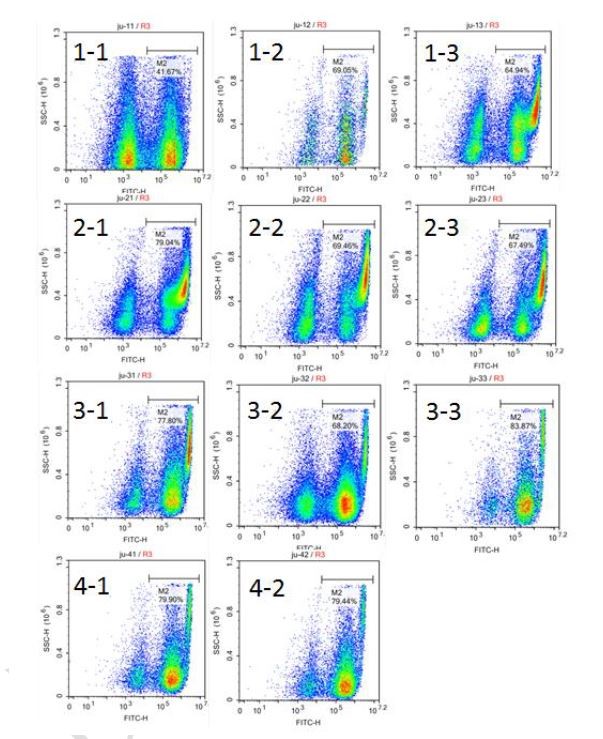

Supplement: Supplement Figure 5 — Macrophage phagocytosis flow cytometer results. [file Image_5.jpeg]
